# Malathion-induced neurological alterations associated with dysregulation of RORγt/STAT3/IL-17 and FOXP3/STAT5/IL-10 pathways: potential neuroprotective effects of BCG

**DOI:** 10.1007/s00210-026-05061-x

**Published:** 2026-02-20

**Authors:** Nora M. Aborehab, Mai A. Abd-Elmawla, Yara M. Aboulmagd, Safaa M. El-Mahdy, Yasmine M. Shahine, Eman M. Osman, Hekmat M. El Magdoub

**Affiliations:** 1 Department of Biochemistry, Faculty of Pharmacy, Ahram Canadian University, Giza, Egypt; 2 Department of Biochemistry, Faculty of Pharmacy, Cairo University, Cairo, 11562 Egypt; 3 Department of Pharmacology and Toxicology, Faculty of Pharmacy, Heliopolis University, Cairo, Egypt; 4 Department of Anatomy & Embryology, Faculty of Medicine, Sohag University, Sohag, Egypt; 5 Department of Microbiology and Immunology, Faculty of Pharmacy, Pharos University, Alexandria, Egypt; 6 Department of Immunology and Allergy Department, Medical Research Institute, Alexandria University, Alexandria, Egypt; 7 Department of Biochemistry, Faculty of Pharmacy, Misr International University, Cairo, Egypt

**Keywords:** Organophosphorus, Malathion, Scopolamine, Bacillus Calmette–Guérin

## Abstract

Despite their effectiveness in agriculture to control a variety of pests, organophosphorus compounds (OPC) such as malathion were linked with neurological dysfunctions and possibly death. The present study aimed to investigate the impact of OPC exposure on neuroinflammation via disrupting the equilibrium between pro-inflammatory (RORγt/STAT3/IL-17/IL-22) and anti-inflammatory (FOXP3/STAT5/IL-10) pathways. The study extended to evaluate the potential of BCG vaccination in alleviating neuroinflammation. Rats were distributed into four groups: control, malathion-intoxicated group, BCG-treated group, and scopolamine-treated group. Behavioral tests and histopathological investigations of the cerebral cortex were done. FOXP3, RORγt, STAT3, and STAT5 were estimated using qRT-PCR. Acetylcholine (Ach), BDNF, IL-10, IL-17, IL-22, BCL2, and BAX were estimated using ELISA, whereas GFAP and IL-1β were estimated via immunohistochemical analysis. The malathion-intoxicated group revealed higher gene expression of RORγt and STAT3, along with lower gene expression of FOXP3 and STAT5, compared with the control group. Moreover, the concentrations of IL-17, IL-22, and BAX were higher, along with lower concentrations of BDNF, IL-10, and BCL2, compared with the control group. Furthermore, GFAP and IL-1β showed marked positive cytoplasmic expression. However, the BCG-treated group reversed all the abovementioned findings. Collectively, the study highlights that malathion induces neuroinflammation via skewing the balance between the proinflammatory (RORγt/STAT3/IL-17/IL-22) and the antiinflammatory (FOXP3/STAT5/IL-10), leading to behavioral fluctations and brain’s histological disruption. This imbalance resulted in cytokine production, neuronal apoptosis, and neurodegeneration. BCG administration alleviates these effects owing to its anti-inflammatory and neuroprotective effects.

## Introduction

Malathion is one of the organophosphorus compounds (OPC) that are commonly used in agriculture to combat pests (Sharma and Bansal 2021). In spite of efficacy, malathion wide application was associated with various health hazards. Malathion inhibits acetylcholinesterase, resulting in marked increase of acetylcholine levels at the synaptic junctions (Patlolla,, et al. 2015). Malathion’s subchronic exposure gives rise to elevated oxidative insults, immunological irregularities, and neuroinflammation. Ample evidence revealed that chronic malathion exposure leads to elevated levels of pro-inflammatory cytokines such as interleukin (IL) −6 and IL-1β (Cui et al. 2024), together with microglial activation and dopaminergic neurodegeneration (Ahmed, et al. 2017; El-Harouny, et al. 2017). Moreover, exposure to malathion was depicted to affect cognitive function, which is manifested by memory deficits and Alzheimer-like symptoms (Cui et al. 2024; Narasimhamurthy et al. 2024). Importantly, OPC exert immunotoxic effects that disrupt immune system function through various mechanisms, leading to immune dysregulation.

Among the primary immunological modulators are the transcription factors retinoic acid-related orphan receptor gamma-t (RORγt) and forkhead box P-3 (FOXP3). RORγt is required for the differentiation of T helper (Th) 17 cells (Kumar et al. 2021). It drives the expression of Th17-associated cytokines, such as IL-17 and IL-22, thus fostering the pro-inflammatory responses. The signal transducer and activator of transcription (STAT)3 is a critical transcription factor responsible for the activation of RORγt and the differentiation and function of Th17 cells (Kumar et al. 2021; Wu et al. 2022; Chang et al. 2019). On the other side, FOXP3 is a transcription factor that is crucial for the differentiation of T regulatory cells (Tregs), which stimulated the release of anti-inflammatory cytokines like IL-10. In addition, STAT5 promoted the transcription of FOXP3, thereby facilitating Treg development and maintenance (Passerini et al. 2008; Wang et al. 2008; Tsuji-Takayama et al. 2008). According to FOXP3 and RORγt, the direction of naïve CD4⁺ T cells differentiation may be directed towards Th17 or Tregs. As well, perturbations of the FOXP3-RORγt axis may result in immune dysregulation and thus various immune-mediated diseases. Since OPC could influence immunological responses, it is expected to modulate FOXP3 and RORγt expression. However, the exact mechanisms are still not fully defined.

Bacillus Calmette–Guérin (BCG), a live-attenuated vaccine that was designed for tuberculosis prevention, has drawn interest owing to its immunomodulatory and anti-inflammatory effects. BCG possessed broad immunomodulatory effects, enhancing immune defense against various infections, certain malignancies, and inflammatory conditions (Korkes et al. 2021; Morrison et al. 2023). BCG vaccination exerts its regulatory immune responses by upregulating FOXP3 expression and stimulating the anti-inflammatory cytokines like IL-10 (Bamberger and Landreth 2002; Jurczak and Druszczynska 2025). BCG attenuated the pro-inflammatory Th17 responses associated with RORγt and IL-17 (Yedke and Kumar 2024). Additionally, BCG has demonstrated neuroprotective effects in preclinical models of multiple sclerosis and other neurological diseases, via regulating the microglial and astrocytic activation (Cossu et al. 2022). Owing to its well-documented safety and immunoregulatory capabilities, BCG may be a promising therapeutic approach for immune dysregulation and neuroinflammatory diseases similar to those that occur in OPC neurotoxicity.

Overall, the current work aimed to elucidate the effect of malathion-induced neuroinflammation by investigating its interplay in disrupting the pro-inflammatory (RORγt/STAT3/IL-17/IL-22), anti-inflammatory (FOXP3/STAT5/IL-10) pathways, and the ongoing apoptotic processes. Furthermore, the research was expanded to evaluate the potential of BCG vaccination in alleviating neuroinflammation through the modulation of these altered immune responses. To provide a comparative framework, scopolamine was employed as an anticholinergic agent that could counteract neurological manifestations associated with OPC toxicity.

## Materials and methods

### Animals

Thirty-two male Wistar albino rats (120–150 g) were obtained from the animal colony of the Faculty of Pharmacy-Cairo University (Cairo, Egypt). The rats were acclimated to controlled environmental conditions (25 °C, 55% humidity, 12-h light/dark cycle) with unrestricted food and water access (standard chow diet). Rats were housed in fiber cages at the Cairo University Faculty of Pharmacy animal facility and left for a 1-week adaptation period. All efforts were made to minimize animal suffering. All procedures of experimental animals were carried out after approval by the Research Ethics Committee for Experimental Studies of the Faculty of Pharmacy, Cairo University, Cairo, Egypt, with approval number: BC (3870).

### Chemicals and drugs

Malathion was purchased from El Nasr CCo. (Egypt). The BCG vaccine was purchased from VACSERA, Cairo, Egypt. Scopolamine was obtained from Boehringer Ingelheim (Egypt). Any other chemicals and reagents used were of high analytical grade.

### Experimental design

**Fig. 1:**
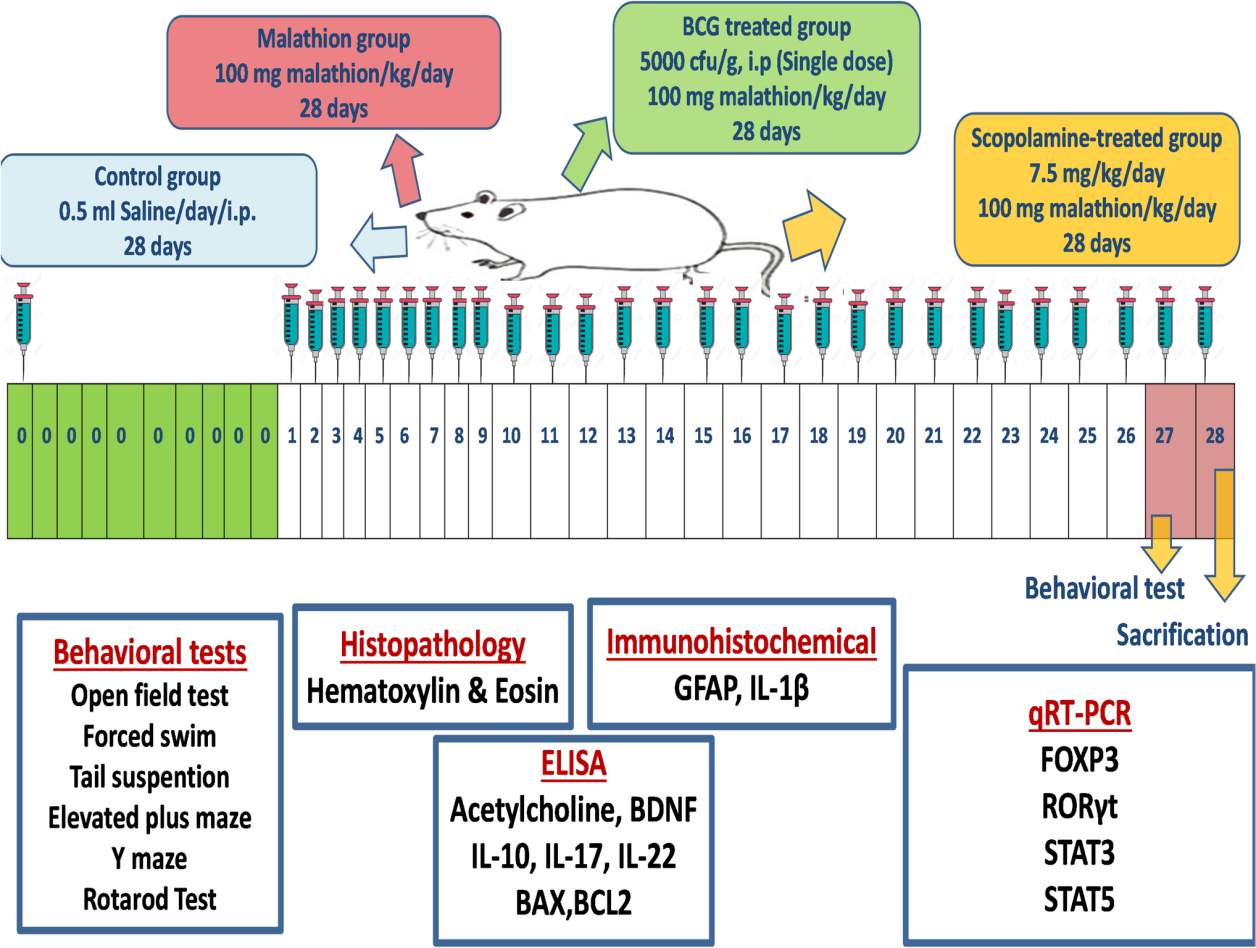
Schematic representation of the study design. BAX, the BCL-2-associated X protein; BCG, Bacillus Calmette–Guérin; BCL2, B-cell leukemia/lymphoma 2 protein; BDNF, brain-derived neurotrophic factor; FOXP3, forkhead box P3; GFAP, glial fibrillary acidic protein; IL, interleukin; RORγt, retinoic acid-related orphan receptor; STAT, signal transducer and activator of transcription

Rats were randomly distributed into four groups, each group containing eight rats, and treated as shown in Fig. 1.

- Control group: Rats were administered 0.5 ml saline (i.p.) daily for 28 days.
- Malathion group: Rats were administered malathion (i.p.) at a dose of 100 mg/kg daily for 28 days (Reus et al. 2008).
- BCG-treated group: Rats received a single injection of the BCG vaccine (5000 cfu/g, i.p.). Ten days later, malathion was administered at a dose of 100 mg/kg/day for 28 consecutive days (Reus et al. 2008; Senousy et al. 2022).
- Scopolamine-treated group: Rats received scopolamine (i.p.) at a dose of 7.5 mg/kg/day, followed by malathion (100 mg/kg/day, i.p.) one hour later, for 28 consecutive days (Reus et al. 2008; Lutz et al. 2000).

On the 27th day, behavioral tests were carried out. Rats were sacrificed on the 28th, and a portion of the cerebral cortex specimens was dissected out in full depth and kept in 10% neutral buffered formalin for histopathological examination, while the other portion was stored at (− 80 °C) for molecular and biochemical analysis.

### Behavioral experiments

Six behavioral tests were conducted by an observer who was blinded to the animals’ treatment groups.

#### Open field test

The open field test is a widely used assessment for evaluating emotional state and anxiety in rats. Each rat was placed in the center of the apparatus, and its movements were tracked, including the immobility time and the number of squares crossed in the peripheral area within the 5-minute observation period (Brain 1999).

#### Forced swim test

Rats were individually placed in transparent cylinders (40 × 18 cm) filled with 15 cm of water at 25 °C. The immobility time of the rat was calculated within the 5-min observation period (Bogdanova et al. 2013).

#### Tail suspension test

In this test, rats were suspended 58 cm above the floor using adhesive tape, with a platform positioned 15–20 cm below to minimize the weight on their tails. The total immobility duration was recorded over 5 min (Chermat et al. 1986).

#### Elevated plus maze

The elevated plus maze is made of wood, consists of two open arms (50 × 10 cm) and two closed arms (50 × 10 × 40 cm) with an open roof, and is arranged in a cross shape. The maze is positioned 50 cm above ground. At the beginning of each 5-min trial, rats were placed at the maze’s center, facing an open arm, and the time spent in closed and opened arms were registered (Mar et al. 2000).

#### Y-maze test

The Y-maze test was conducted with arms labeled A, B, and C. The rats were initially placed in arm A, opposite to the maze’s center, and allowed to explore the uninterrupted maze for 5 minutes. The rat’s spontaneous alternations were tracked and recorded (Lutz et al. 2000).

#### Rotarod test

Rats were acclimated to a rotating rod at a low speed (4 rpm) for 1 min. Then, they underwent three consecutive trials, with 30-min breaks in between, using an accelerated speed program. The time for each rat to fall off the rod was recorded. The average time to fall from the three trials was calculated for statistical analysis and used to determine the average latency to fall (in seconds) for each group (Pritchett and Mulder 2003).

### Histopathologic examination

Brain tissue sections (3–4 mm thick) were fixed in 10% neutral buffered formalin. They were then dehydrated using ethanol, cleared with xylene, and embedded in paraffin. Paraffin blocks were sliced into 4–6 μm-thick sections using a microtome. Sections were stained with hematoxylin and eosin (H&E) to examine general tissue structure. H&E-stained sections were analyzed using a Leica microscope (CH9435 Hee56rbrugg, Leica Microsystems, Switzerland) (Bancroft and Gamble 2008).

### Immunohistochemical assessment and its quantitative scoring

Immunohistochemistry was performed on paraffin-embedded tissue sections using the avidin-biotin-peroxidase complex (ABC) method. The sections were incubated with specific antibodies: mouse glial fibrillary acidic protein (GFAP) monoclonal antibody (Elabscience Cat# E-AB-70205, Clone: 2B12F1, Dilution: 1:500) and Rabbit Anti-IL-1beta antibody, Clone [RM1009] (Abcam Cat# ab283818, Dilution: 1:500). The ABC technique was applied using Vectastain ABC-HRP kit, Vector Laboratories. Marker expression was identified with peroxidase and stained with diaminobenzidine (DAB, produced by Sigma) to distinguish the antigen-antibody complex. Negative controls were included by substituting non-immune serum for primary or secondary antibodies. Immunostained sections were examined and photographed using a Leica microscope (CH9435 Hee56rbrugg) at various magnifications. (Leica Microsystems, Switzerland). In each section of the groups studied, six high-power fields (×400) with positive brown immunostaining were selected for analysis. The area percentage of GFAP and IL-1β staining was quantified using the Leica QWin 500 image analyzer system (England). This system consists of a Leica microscope, a color video camera, a monitor, and a computer with Leica QWin 500 software, which analyzes the images.

### Determination of ACh, brain-derived neurotrophic factor (BDNF), IL-10, IL-17, IL-22, BCL2, and BAX using ELISA immunoassay

Commercial ELISA kits were used to assess the concentrations of ACh, BDNF, and IL-10 supplied from Elabscience, USA (Catalog no: E-EL-0081), Invitrogen, USA (Catalog no. ERBDNF), and Cusabio, USA (Catalog no. CSB-E04595r), respectively. IL-17 (Catalog no. MBS2022678), IL-22 (Catalog no. MBS285806), and BCL2 (Catalog no. MBS2515143) were supplied by MyBioSource, USA. BAX was measured using Nova kit (Catalog no. NBP2-69938). All procedures were done according to the manufacturer’s guidelines.

### Determination of FOXP3, RORγt, STAT3 and STAT5 using reverse transcription-polymerase chain reaction (qRT-PCR)

Total RNA was isolated from the cerebral cortex homogenates using a total RNA tissue extraction kit (Catalog no. R2050) (Zymo Research, California, USA) according to the manufacturer’s instructions. Then, reverse-transcribed using RT-PCR kit (Catalog no. WF-1020500X) (Willowfort, UK). Quantitative RT-PCR was performed using SYBR Green qPCR Kit (Catalog no. WF1030800X) (Willowfort, UK). The sequences of the primers are listed in Table 1.

**Table 1:** List of primers

Gene	Forward	Reverse
FOXP3	5’-TCC CTT CCG AGA TGG T-3’	5’-GCT GGA TCC ACT TGA GGT-3’
RORγt	5’-CCC ACA GCA CAG TTT CAA-3’	5’-GCA GGT GGC TGA GGT TGT-3’
STAT3	5’-TCA GGT CAA GAC CAG GTT-3’	5’-GCT GCA GGT AGT GGT GTA-3’
STAT5	5’-GCA GAG GAC CAG AAC CAG-3’	5’-TCA GGT CAA GAC CAG GTT-3’
Β**-**actin	5’-CCACCAGTTCGCCATGGAT-3’	5’-GTTGGTGACAATGCCGTGTT-3’

### Statistical analysis

The results are depicted as mean ± standard error (SE). Normality was checked by the Shapiro-Wilk test. One-way ANOVA and Tukey’s multiple comparison tests were used in the statistical investigations within groups. A *p*-value of < 0.05 was considered statistically significant. The data were generated using GraphPad Prism software (Inc. V5, USA).

## Results

### Effects of BCG and scopolamine on behavioral alterations in malathion-intoxicated rats

#### Elevated plus maze test

**Fig. 2:**
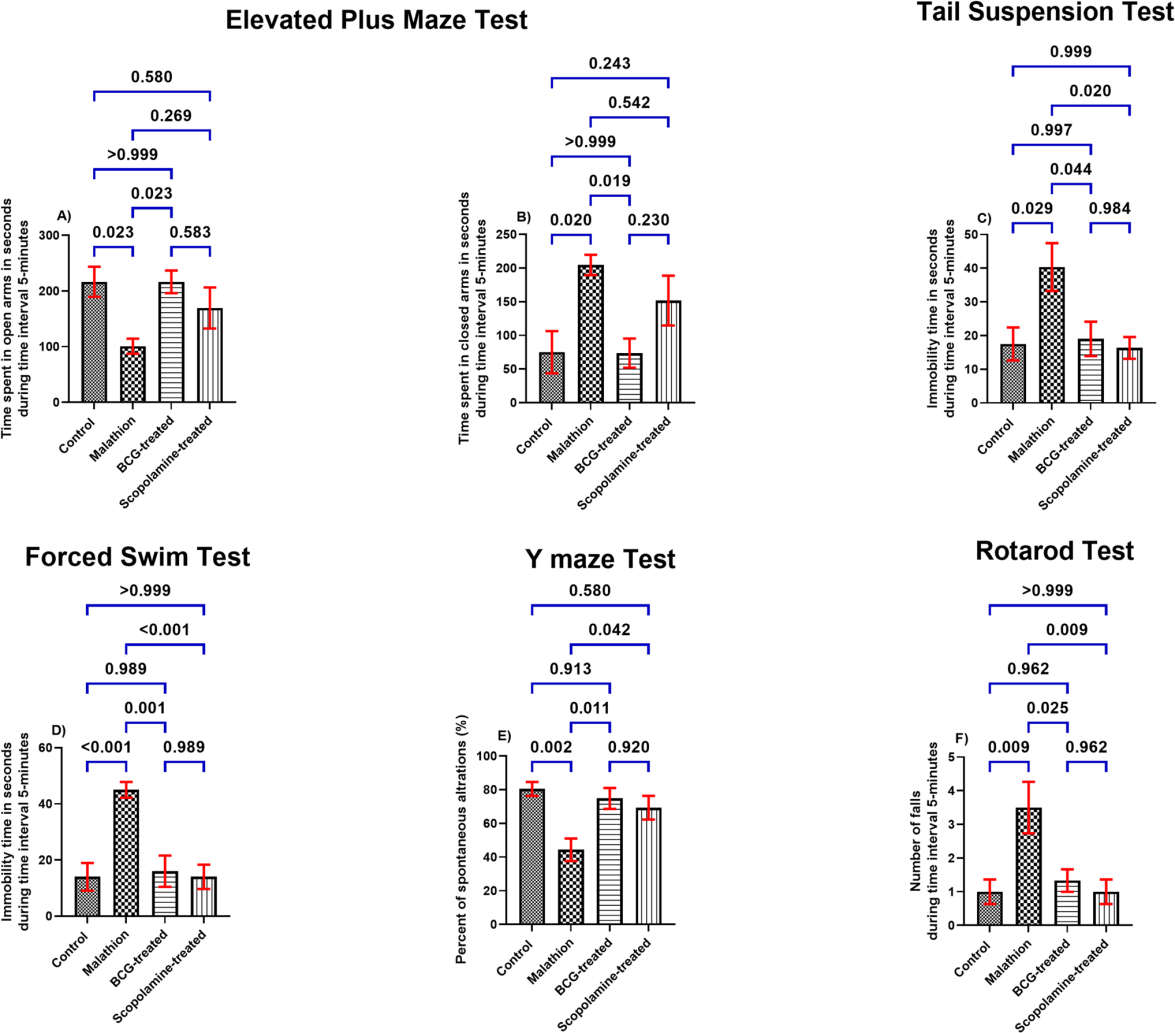
Effects of BCG and scopolamine on the behaviors of rats. **A**, **B** The elevated plus maze test, **C** the tail suspension test, **D** the forced swim test, **E** the Y-maze test, and **F** the rotarod test. Data are shown as mean ± SE. Statistical investigations were done by the ANOVA test and then Tukey’s post hoc test. BCG, Bacillus Calmette–Guérin

The malathion-intoxicated group showed a marked reduction in the time consumed in opened arms and a considerable increase in the time consumed in the closed arms versus the control group. The BCG-treated group showed less anxious behaviors, where the time consumed in the opened arm was markedly higher and the time consumed in the closed arm was markedly lower relative to the malathion-intoxicated group (Fig. 2 A and B).

#### Tail suspension test

Compared with the control group, the malathion-intoxicated group showed a marked elevation in immobility time. On the contrary, the BCG-treated group demonstrated a noticeably shorter immobility time compared with the malathion-intoxicated group (Fig. 2C).

However, no significant difference was revealed between the scopolamine-treated group and the malathion-intoxicated one in terms of the elevated plus maze test and tail suspension test.

#### Forced swim test

The immobility time was prolonged in the malathion-intoxicated group compared with the control group, whereas the BCG and scopolamine-treated groups exhibited a marked reduction in the immobility time compared with the malathion-intoxicated group (Fig. 2D).

#### Y-maze test

In the malathion-intoxicated group, there were marked memory impairments, where a significant decrement in the percentage of alternations was shown relative to the control group. The BCG and scopolamine-treated groups possessed noticeable improvement in memory, where the percentage of alternations was higher relative to the malathion-intoxicated group (Fig. 2E).

#### Rotarod test

This test revealed that the number of falls was higher in the malathion-intoxicated group compared with the control group. The BCG and scopolamine-treated groups showed a lower number of falls relative to the malathion-intoxicated group (Fig. 2F).

#### Open field test

**Fig. 3:**
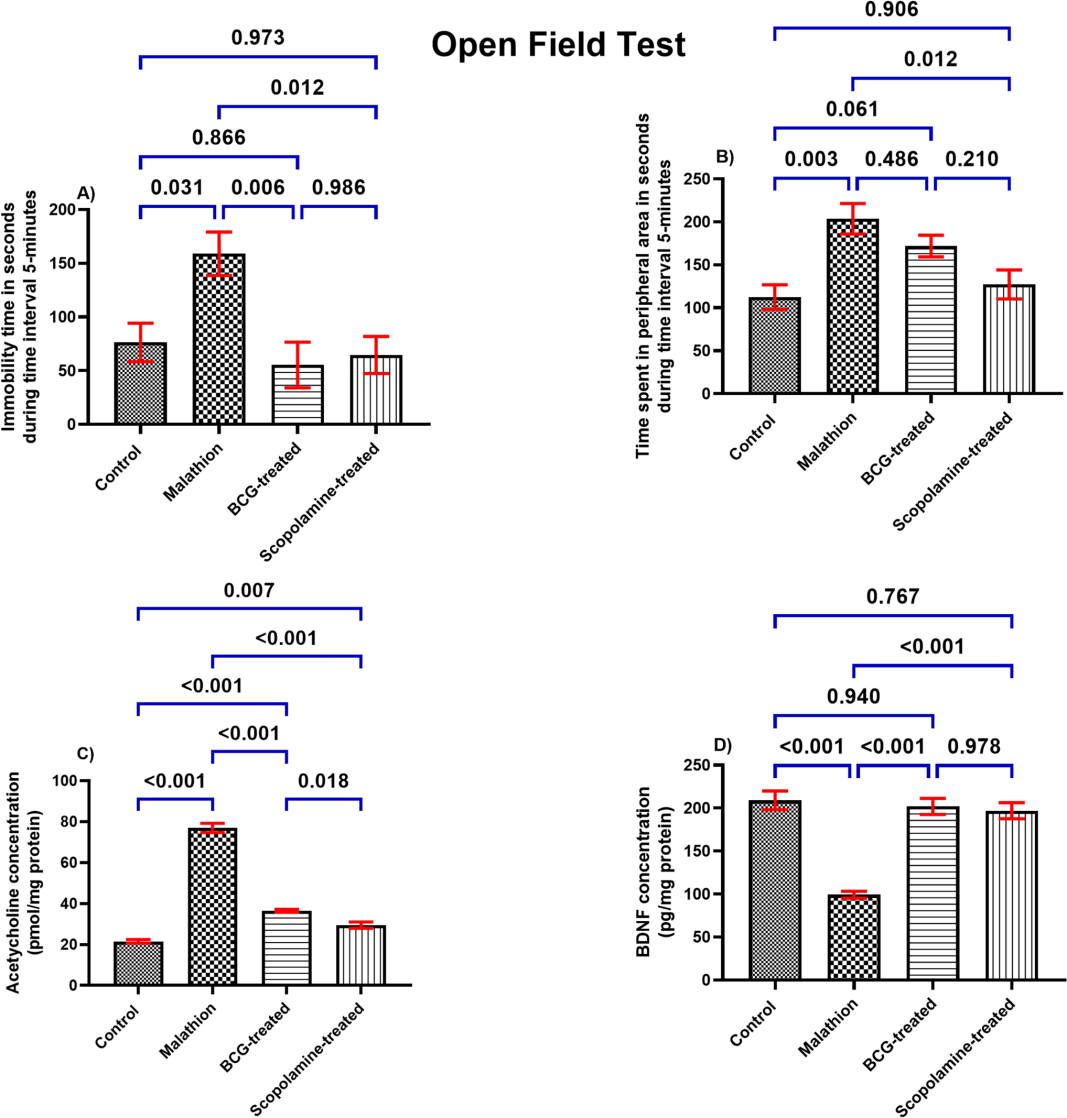
Effects of BCG and scopolamine on the behaviors of rats. **A**, **B** The open field test and the concentrations of **C** acetylcholine and **D** BDNF. Data are shown as mean ± SE. Statistical investigations were done by the ANOVA test and then Tukey’s post hoc test. BCG, Bacillus Calmette–Guérin; BDNF, brain-derived neurotrophic factor

The immobility time and time spent in the peripheral area were higher in the malathion-intoxicated group compared with the control group. On the other hand, the immobility time and time spent in the peripheral area were lower in the BCG and the scopolamine-treated groups compared with the malathion-intoxicated group (Fig. 3A and B).

### Effects of BCG and scopolamine on the cerebral contents of ACh and BDNF in malathion-intoxicated rats

As expected, the ACh content was markedly elevated in the malathion-intoxicated group compared with the control group, whereas the ACh content was markedly lower in the BCG and scopolamine-treated groups compared with the malathion-intoxicated group. It is noteworthy that the content of ACh was significantly lower in the scopolamine-treated group compared to the BCG-treated group. In parallel, the BDNF content was significantly decreased in the malathion-intoxicated group compared with the control group. Meanwhile, it was significantly higher in the BCG and scopolamine-treated groups compared with the malathion-intoxicated group. Moreover, there were statistically significant differences between the control group and both the BCG and the scopolamine-treated groups (Fig. 3C and D).

### Effects of BCG on the cerebral histopathological changes in malathion-intoxicated rats

**Fig. 4:**
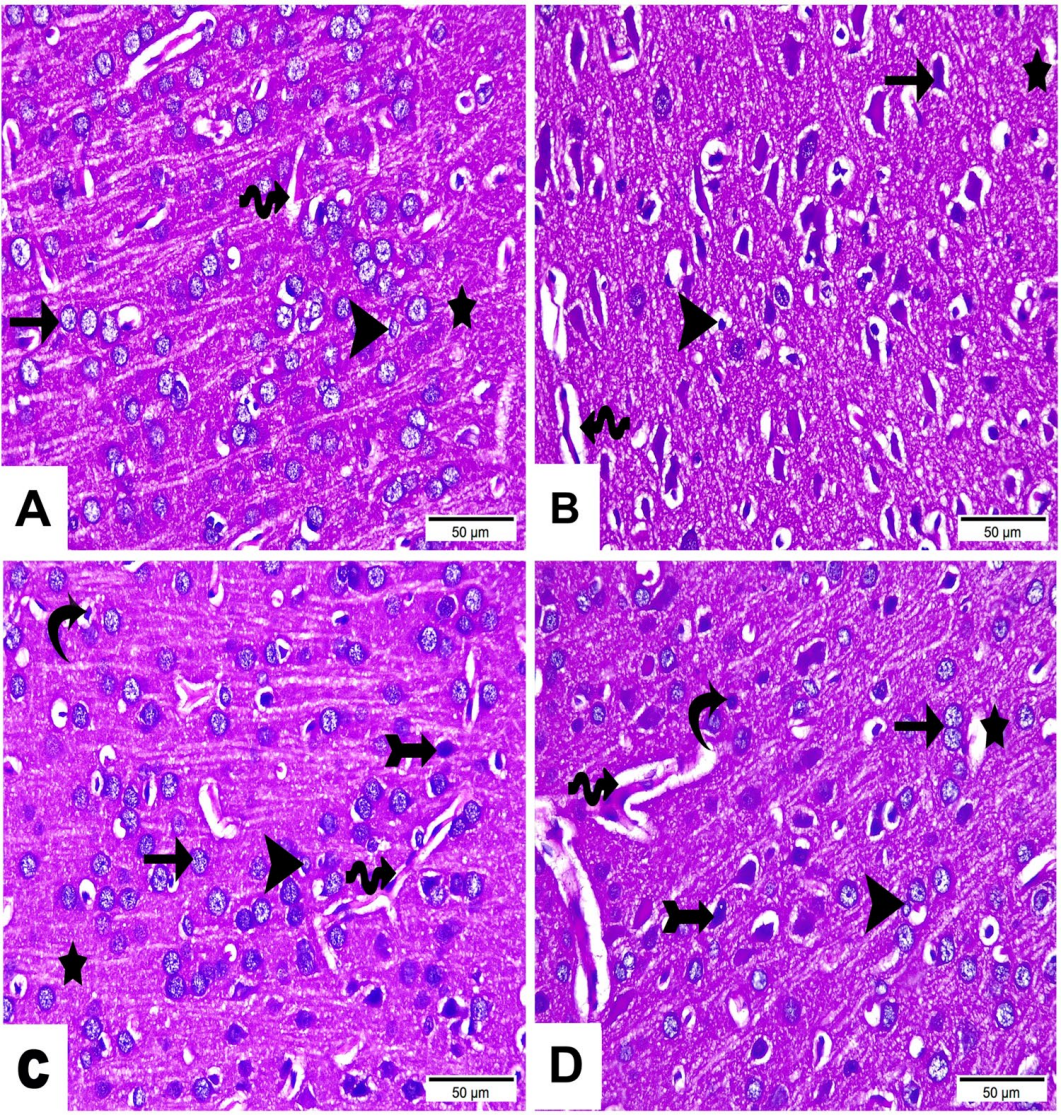
Effects of BCG and scopolamine on the hematoxylin and eosin stain histopathological changes of the rat’s cerebral cortex (scale bar 50 µm). **A** Control group, **B** malathion-intoxicated group, **C** BCG group, and **D** scopolamine group. BCG, Bacillus Calmette–Guérin

The cerebral cortex section of the control group revealed normal assembly of neurons with light vesicular nuclei, prominent nucleoli (arrow). Neuroglial cells are specified with round central nuclei between neurons (arrowhead). An intense network of linked nerve fibers and their branches and synapses, concurrently with glial filaments, constructed the neuropil (star). Blood capillaries are also distinguished along the cortical area (wave arrow) (Fig. 4A). On the other hand, the malathion-intoxicated group disclosed serious degenerative changes. Neurons emerged with shrunken, hyperchromatic, and deep basophilic nuclei (arrow). Gliosis was detected along the tissue with some neuroglia cells displaying vacuolated cytoplasm and apoptotic nuclei (arrowhead). Notice vacuolations in between neurons (star) plus dilated congested blood capillaries (wave arrow) were also spotted (Fig. 4B).

Regarding the BCG-treated group, the cerebral cortex section showed highlighted obvious improvement in tissue structure. Neurons were detected mainly like normal with light vesicular prominent nuclei (arrow), while a few neurons had apoptotic shrunken hyperchromatic nuclei (arrowhead). Neuroglia cells demonstrated vacuolated cytoplasm and apoptotic nuclei (curvy arrow) while most others had a regular appearance (arrowhead). Neuropil observed with intact structure (star) except few blood capillaries still seen in a dilated and congested look (wave arrow) (Fig. 4C). In scopolamine-treated groups, the cerebral cortex section showed moderate enhancement in tissue structure. Neurons characterized with some apoptotic shrunken hyperchromatic ones (arrow with tail) and others detected with light vesicular prominent nuclei (arrow). Glia cells exhibited a regular shape (arrowhead) and others with apoptotic nuclei (curvy arrow). Neuropil detected with some vacuolations (star) as well as obvious dilatation and congestion of blood capillaries (wave arrow) (Fig. 4D).

### Effects of BCG and scopolamine on the GFAP antibody’s reaction in the cerebral cortex of malathion-intoxicated rats

**Fig. 5:**
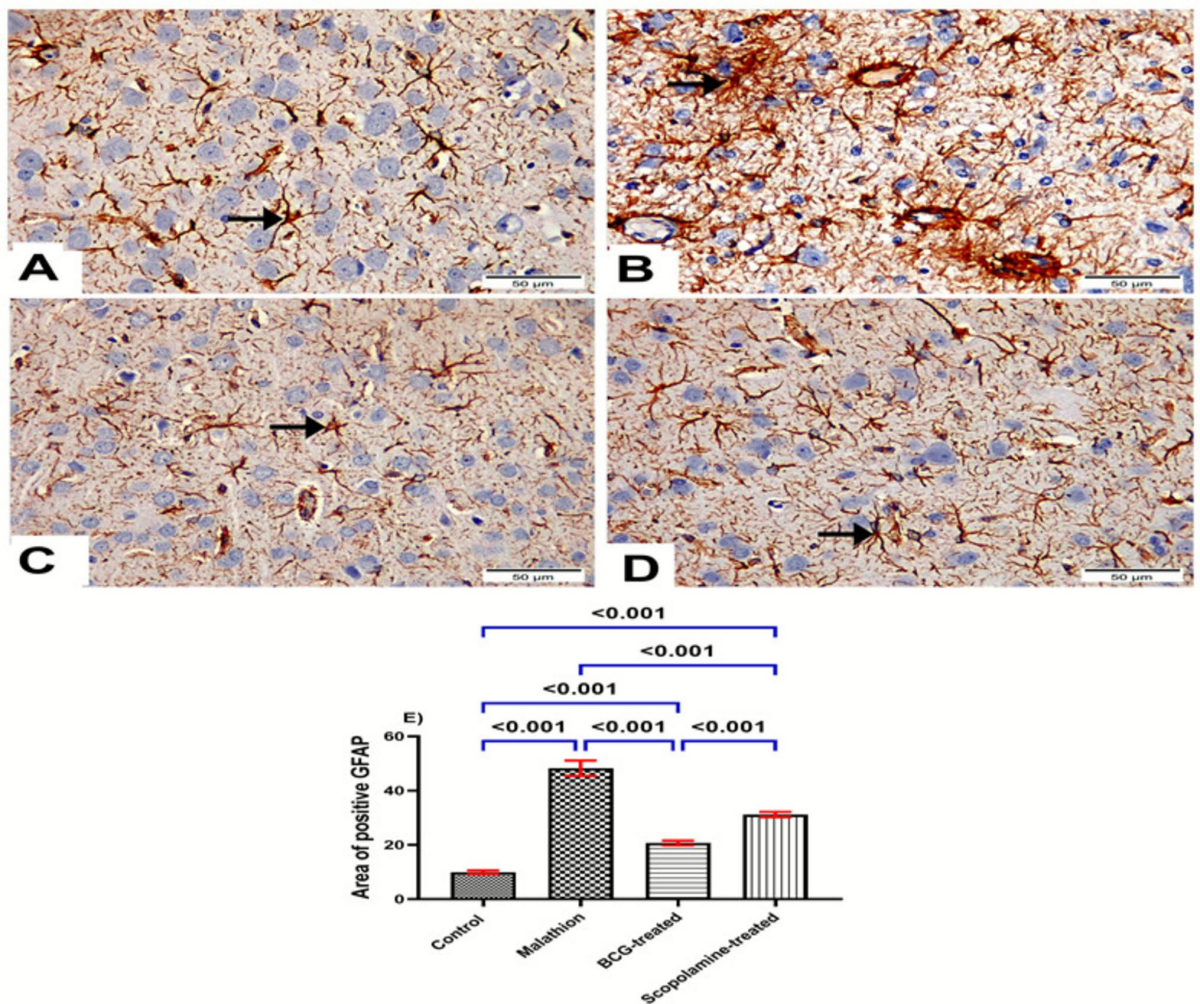
Effects of BCG and scopolamine on the reactivity of GFAP antibodies in the cerebral cortex of rats (scale bar 50 µm). **A** Control group, **B** malathion-intoxicated group, **C** BCG-treated group, and **D** scopolamine-treated group. **E** Results are presented as mean ± SE. Statistical investigations were done by the ANOVA test and then Tukey’s post hoc test. BCG, Bacillus Calmette–Guérin; GFAP, glial fibrillary acidic protein

The cerebral cortex section from the control group showed scarce cytoplasmic GFAP reactivity along neuroglia cells (arrow), whereas those of the malathion-intoxicated rats exhibited intense positive cytoplasmic GFAP reaction along neuroglia cells (arrow) with a significant difference compared with the control group. The cerebral cortex section from the BCG-treated group revealed a few positive cytoplasmic GFAP reactions along neuroglial cells (arrow), with significant differences relative to the control group and the malathion-intoxicated group. The scopolamine-treated group presented moderate positive cytoplasmic expression of GFAP along neuroglial cells (arrow), with significant differences from the control group, the malathion-intoxicated group, and the BCG-treated group (Fig. 5A–D).

### Effects of BCG on RORγt, STAT3, IL-17, and IL-22 in the cerebral cortex of malathion-intoxicated rats

**Fig. 6:**
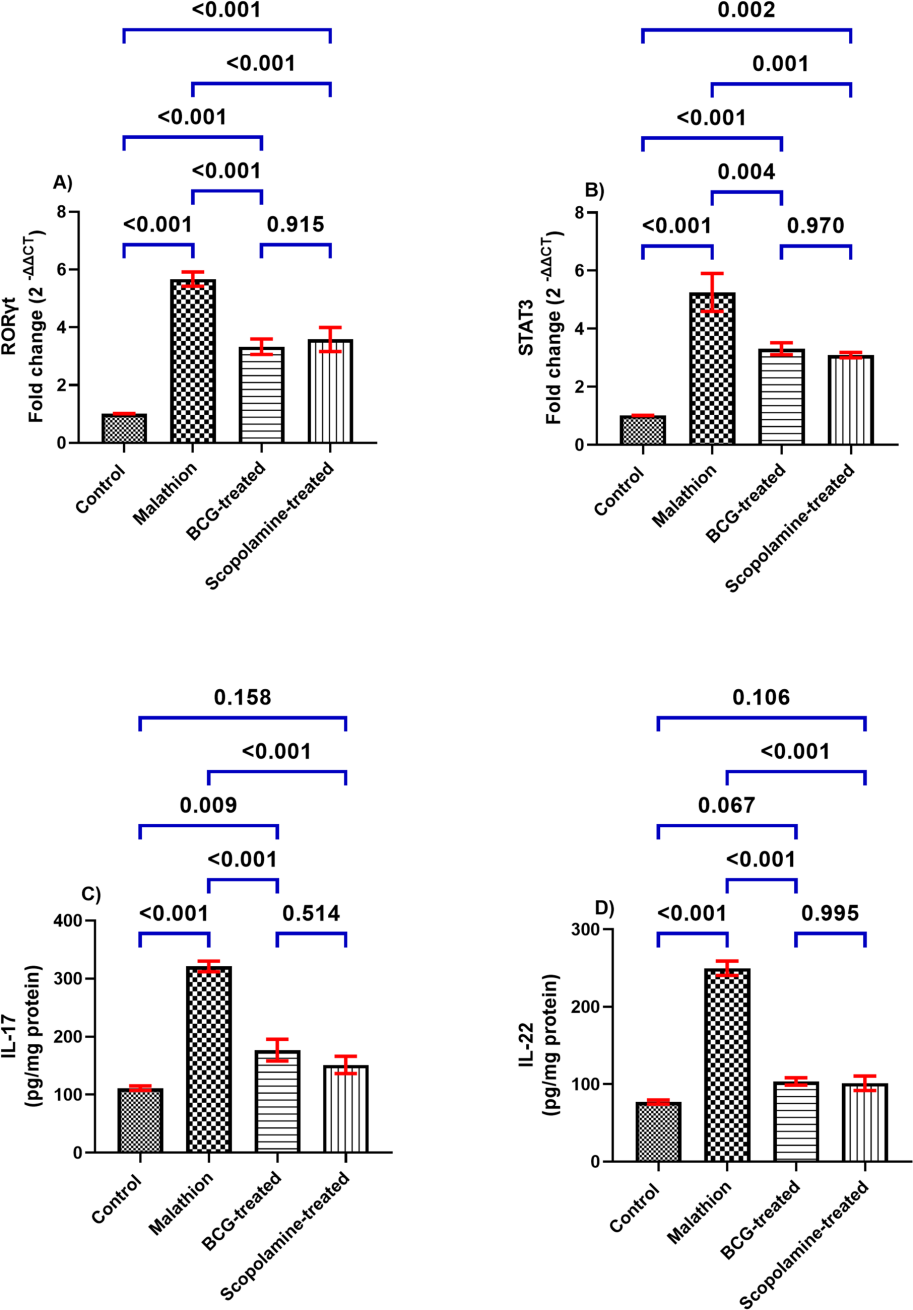
Effects of BCG and scopolamine on the gene expressions of **A** RORγt, **B** STAT3, and the protein content of **C** IL-17 and **D** IL-22 in the rat’s cerebral cortex. Results are presented as mean ± SE. Statistical investigations were done by the ANOVA test and then Tukey’s post hoc test. IL, interleukin; RORγt, retinoic acid-related orphan receptor; STAT, signal transducer and activator of transcription

The gene expression of RORγt and STAT3 were significantly upregulated in the malathion-intoxicated group compared with the control group, whereas their expression levels were significantly downregulated in the BCG and the scopolamine-treated groups compared to the malathion-intoxicated group. At the same time, the concentrations of IL-17 and IL-22 were significantly increased in the malathion-intoxicated group compared with the control group, whereas their concentrations were lower in both the BCG and the scopolamine-treated groups compared to the malathion-intoxicated group. Noteworthy is that both the BCG and the scopolamine-treated groups showed comparable results (Fig. 6A–D).

### Effects of BCG on FOXP3, STAT5, and IL-10 in the cerebral cortex of malathion-intoxicated rats

**Fig. 7:**
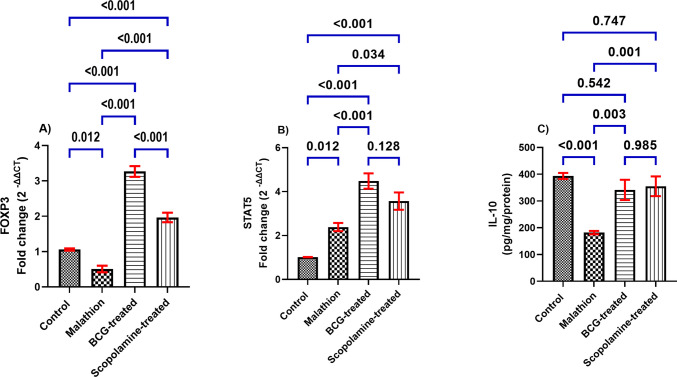
Effects of BCG and scopolamine on the gene expressions of **A** FOXP3, **B** STAT5, and the protein content of **C** IL-10 in the rat’s cerebral cortex. Results are presented as mean ± SE. Statistical investigations were done by the ANOVA test and then Tukey’s post hoc test. BCG, Bacillus Calmette–Guérin; FOXP3, forkhead box P3; IL, interleukin; STAT, signal transducer and activator of transcription

The gene expression of FOXP3 and STAT5 were significantly downregulated in the malathion-intoxicated group compared with the control group, whereas their expression levels were significantly upregulated in the BCG and the scopolamine-treated groups compared with the malathion-intoxicated group. At the same time, the concentration of IL-10 was significantly decreased in the malathion-intoxicated group compared with the control group, whereas its concentration was elevated in both the BCG and the scopolamine-treated groups compared to the malathion-intoxicated group. Noteworthy, the elevation of FOXP3 expression was higher in the BCG-treated group compared to the scopolamine-treated group, whereas no significant differences were noticed in terms of STAT5 and IL-10 (Fig. 7A–C).

### Effects of BCG on FOXP3/RORγt, STAT3/STAT5, and IL-17/IL-10 ratios in the cerebral cortex of malathion intoxicated rats

**Fig. 8:**
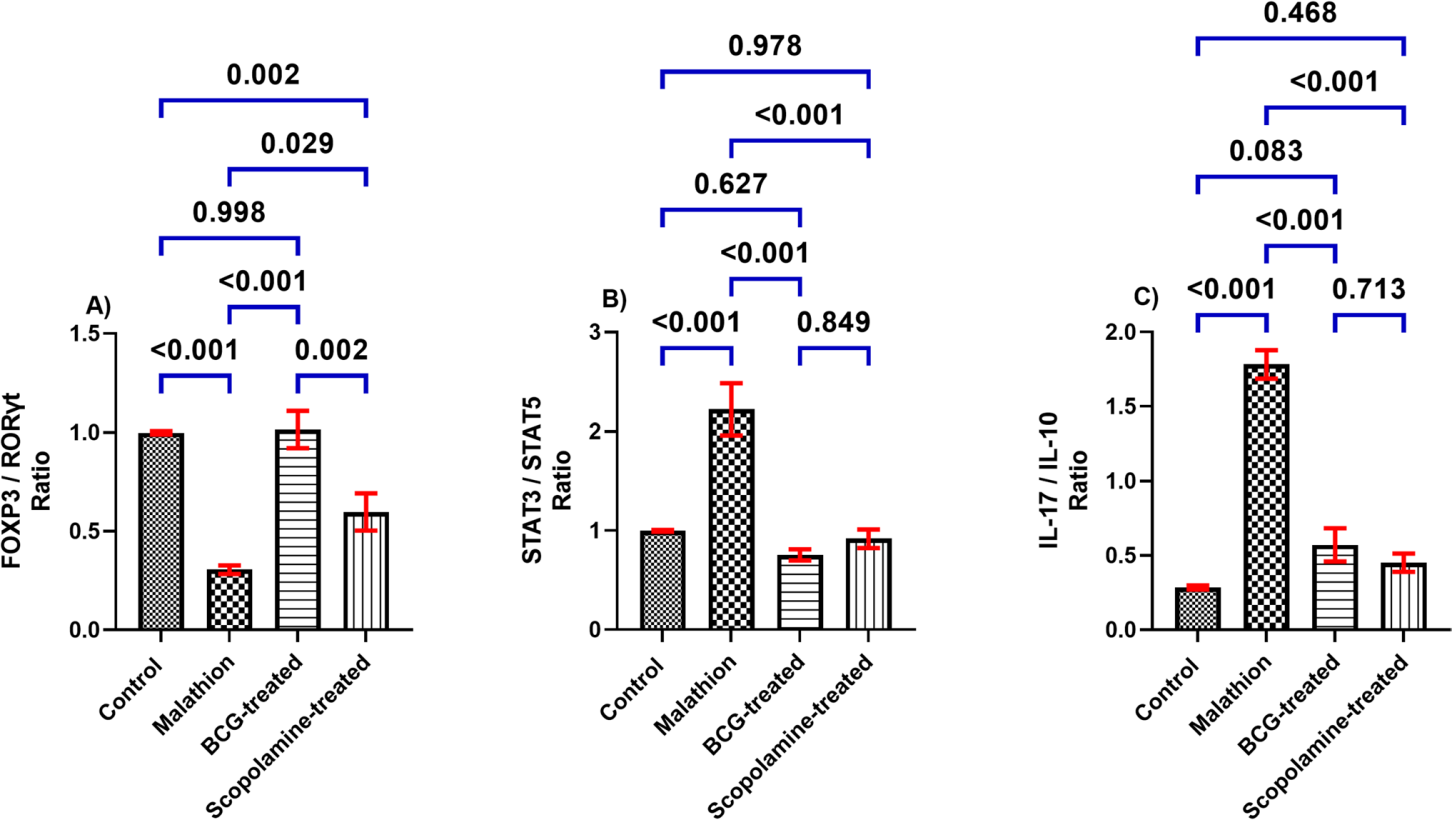
Effects of BCG and scopolamine on the ratios **A** FOXP3/RORγt, **B** STAT3/STAT5, and **C** IL-17/IL-10 in the rat’s cerebral cortex. Results are presented as mean ± SE. Statistical investigations were done by ANOVA test and then Tukey’s post hoc test. BCG, Bacillus Calmette–Guérin; FOXP3, forkhead box P3; IL, interleukin; RORγt, retinoic acid-related orphan receptor; STAT, signal transducer and activator of transcription

The FOXP3/RORγt ratio was significantly decreased in the malathion-intoxicated group compared with the control group, and markedly higher in the BCG and the scopolamine-treated groups compared to the malathion-intoxicated group. It is worth noting that the ratio was higher among the BCG-treated group compared to the scopolamine-treated group. On the other side, the STAT3/STAT5 and IL-17/IL-10 were higher in the malathion-intoxicated group compared with the control group, whereas these ratios were markedly lower in the BCG and the scopolamine-treated groups compared to the malathion-intoxicated group (Fig. 8A–C).

### Effects of BCG on the IL-1β antibody’s reaction in the cerebral cortex of malathion-intoxicated rats

**Fig. 9:**
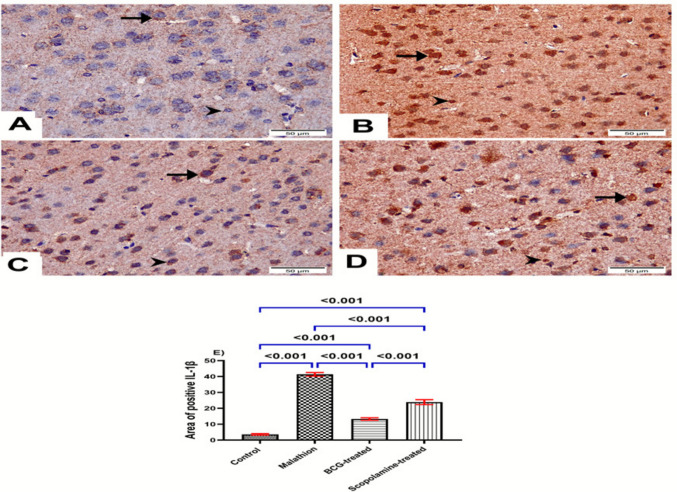
Effects of BCG and scopolamine on the reactivity of IL-1β antibodies in the cerebral cortex of rats (scale bar 50 µm). **A** Control group, **B** malathion-intoxicated group, **C** BCG-treated group, and **D** scopolamine-treated group. **E** Results are presented as mean ± SE. Statistical investigations were done by the ANOVA test and then Tukey’s post hoc test. BCG, Bacillus Calmette–Guérin; IL, interleukin

The cerebral cortex section of the control group demonstrated scarce cytoplasmic IL-1β reactivity along neurons (arrow) and neuroglial cells (arrowhead). The malathion-intoxicated group revealed strong positive nuclear IL-1β reaction along neurons (arrow) and neuroglial cells (arrowhead), with a significant difference from the control group. The cerebral cortex section from the BCG-treated group disclosed a few positive cytoplasmic IL-1β expression along neurons (arrow) and neuroglial cells (arrowhead) with significant differences from the control group and the malathion-intoxicated group. The cerebral cortex section of the scopolamine group revealed a moderate positive cytoplasmic expression of IL-1β along neurons (arrow) and neuroglia cells (arrowhead) with significant differences from the control, the malathion-intoxicated, and the BCG-treated groups (Fig. 9A–D).

### Effects of BCG on BAX, BCL2, and their ratio in the cerebral cortex of malathion-intoxicated rats

**Fig. 10:**
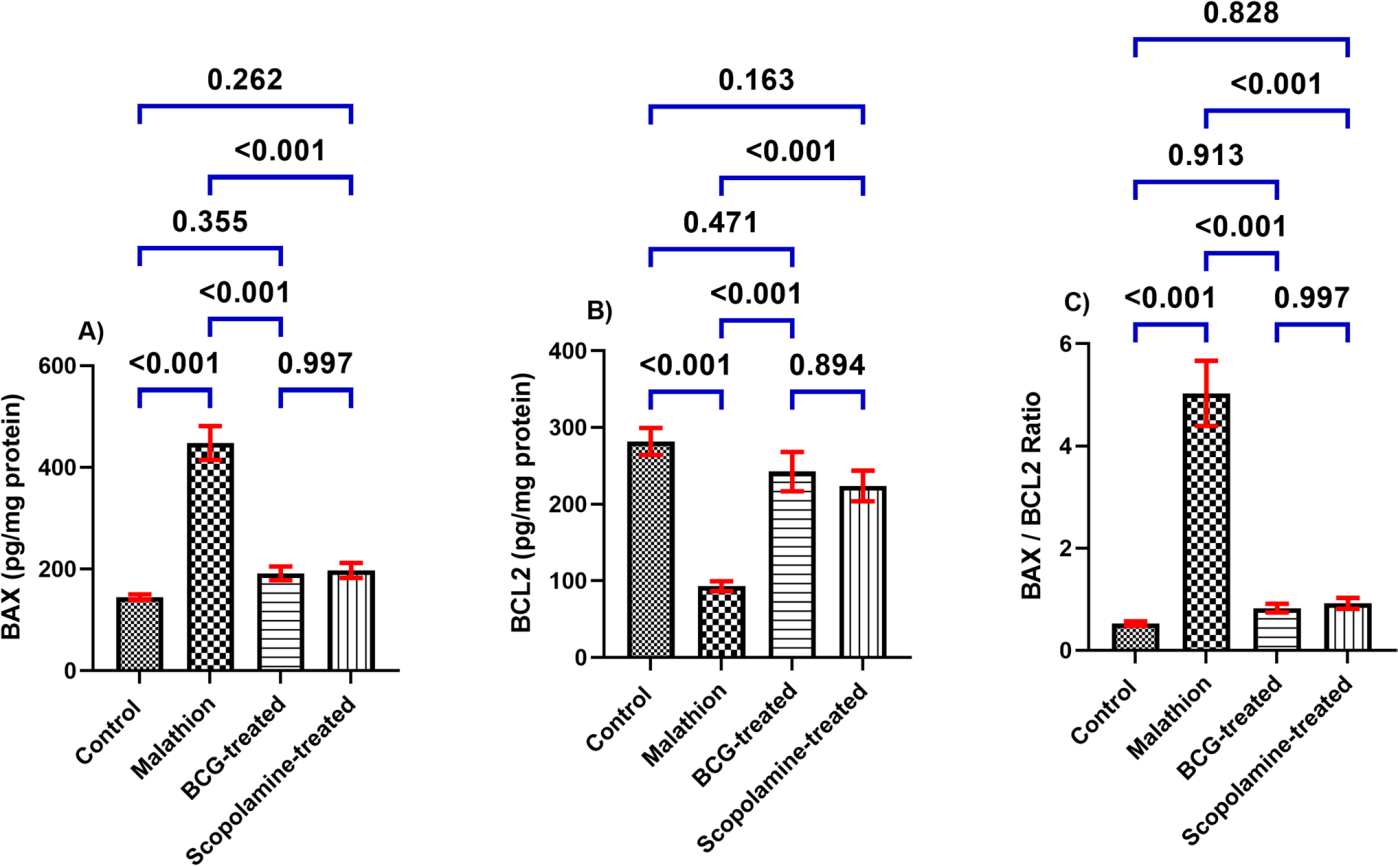
Effects of BCG and scopolamine on the concentrations of **A** BAX, **B** BCL2, and the BAX/BCL2 ratio in the rat’s cerebral cortex. Results are presented as mean ± SE. Statistical investigations were done by the ANOVA test and then Tukey’s post hoc test. BAX, BCL-2-associated X protein; BCG, Bacillus Calmette–Guérin; BCL2, B-cell leukemia/lymphoma 2 protein

The BAX concentration was significantly higher in the malathion-intoxicated group compared with the control group, whereas it was markedly lower in the BCG and the scopolamine-treated groups compared with the malathion-intoxicated group. On the other side, the BCL2 concentration was lower in the malathion-intoxicated group compared with the control group, whereas it was markedly higher in the BCG and the scopolamine-treated groups compared with the malathion-intoxicated group (Fig. 10A and B). It is noteworthy that the apoptotic/antiapoptotic effects of both treatments were similar. In addition, the ratio of BAX/BCL2 was significantly higher in the malathion-intoxicated group compared with the control group, whereas it was markedly lower in the BCG and the scopolamine-treated groups compared with the malathion-intoxicated group (Fig. 10C).

## Discussion

**Fig. 11:**
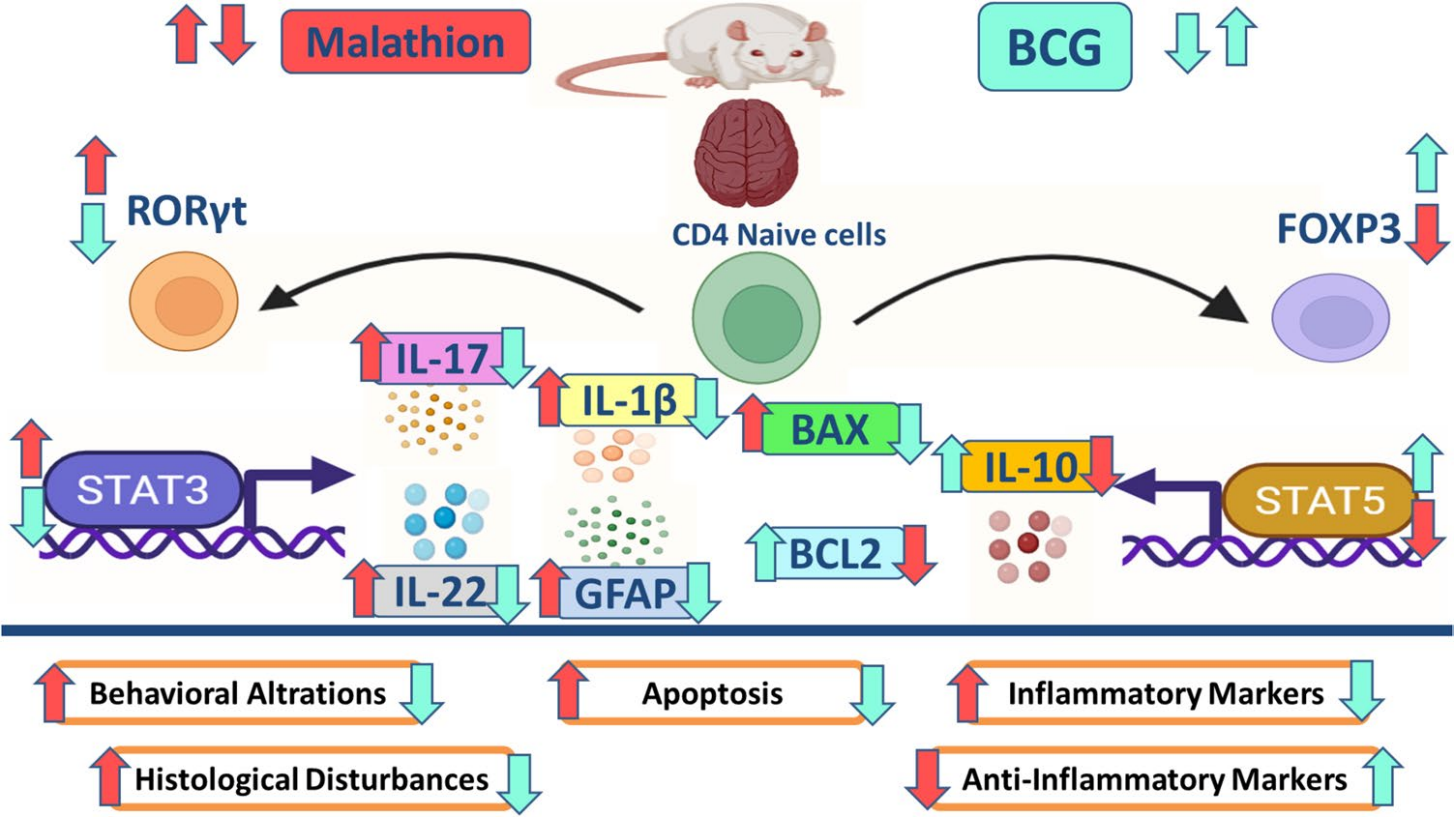
Schematic illustration of malathion-induced neuroinflammation via activation of the RORγt/STAT3/IL-17 axis and suppression of the FOXP3/STAT5/IL-10 regulatory pathway. BCG administration restores immune balance. BAX, the BCL-2-associated X protein; BCG, Bacillus Calmette–Guérin; BCL2, B-cell leukemia/lymphoma 2 protein; BDNF, brain-derived neurotrophic factor; FOXP3, forkhead box P3; GFAP, glial fibrillary acidic protein; IL, interleukin; RORγt, retinoic acid-related orphan receptor; STAT, signal transducer and activator of transcription

Organophosphorus compounds such as malathion are widely used as pesticides and are known to induce neuroinflammation through multiple mechanisms. The primary mechanism of OPC toxicity involves the inhibition of acetylcholine esterase (AChE), leading to overstimulation of the cholinergic receptors (Kumaravel et al. 2024). Ample evidence revealed that OPC alter the number and function of various immune cells, resulting in loss of immune homeostasis, which contributes to the development of neuroinflammation and the subsequent neurological impairments (Bindhani et al. 2022; Andrew and Lein 2021; Camacho-Pérez et al. 2022). Understanding these mechanisms is crucial for developing therapeutic strategies to mitigate the deleterious effects of OPC exposure. The current study was directed to explore the effect of OPC exposure on neuroinflammation via disturbing the proinflammatory (RORγt/STAT3/IL-17/IL-22) and anti-inflammatory (FOXP3/STAT5/IL-10) balance, which subsequently activates further inflammatory cytokines and apoptosis (Fig. 11). Furthermore, the study was broadened to assess the effect of BCG in mitigating neuroinflammation via reshaping the altered immune responses.

The current study revealed that malathion administration induced neurological impairments, which were evidenced by behavioral and histopathological assessments. The behavioral assessments underscored that the malathion-intoxicated group was suffering from depression and anxiety-like symptoms along with cognition and motor impairments. Thoroughly, the anxiety-like symptoms were demonstrated by the shortened time spent in the open arm, along with the prolonged time spent in the closed arm of the elevated plus maze. Anxiety was also demonstrated by the open field test, which revealed that the immobility time and time spent in the peripheral area were higher. The depression-like symptoms were indicated by the prolonged immobility time in the tail suspension test and the forced swim test. The cognitive impairments were also revealed by the Y-maze test, which showed a low percentage of spontaneous alternations. Motor coordination impairments were also indicated by the increased number of falls in the rotarod test. All of these behavioral alterations exhibited by the malathion-intoxicated group reflect the occurrence of neurological disturbances. Previous literature demonstrated that exposure to OPC triggered behavioral perturbations, particularly affecting cognitive and emotional functions (Ullah et al. 2025; Berroug et al. 2024; Zhuo et al. 2024). These cognitive alterations could appear as difficulties in learning, reduced problem-solving abilities, and low academic performance, especially when exposure occurred in the critical periods of neurodevelopment, such as prenatal or in childhood (Khademi et al. 2023).

In continuation of the deleterious effects of malathion, the histological structure of the cerebral cortex showed marked degeneration and marked neuronal and glial pathology. The nuclei of the cortical neurons appeared shrunken, hyperchromatic, and deeply basophilic. The presence of gliosis, manifested by an increase in the reactive glial cells, together with neuroglial cells possessing vacuolated cytoplasm and apoptotic nuclei, thus reflects the reactive astrocyte responses to the injury of the neurons. These histological alterations are similar to those seen in different neurotoxic insults (Morsi et al. 2022; Massoud et al. 2022). In the same context, GFAP was highly expressed in the malathion group, thus reflecting astrocyte activation, which usually exists in neuroinflammation and CNS injury (Santos et al. 2016).

The principal reason underlying OPC toxicities is the inhibition of AChE (Aroniadou-Anderjaska et al. 2023), which was evidenced by high levels of ACh in the malathion-intoxicated group in the present study. Accumulation of ACh at synaptic junctions leads to overstimulation of nicotinic and muscarinic receptors. Such overstimulation can cause a range of symptoms from acute cholinergic crises to chronic neurological disturbances (Aroniadou-Anderjaska et al. 2023; Figueroa-Villar et al. 2021). The excessive accumulation of ACh could predispose to receptor desensitization, leading to irreversible neurological deficits, as manifested in the present study by the marked decrement of BDNF content in the brain, pinpointing the neurological impairments and cognitive delay. In fact, BDNF exerts a potential function in neuronal development, differentiation, and synaptic plasticity (Rodríguez-Carrillo et al. 2024; Dorri et al. 2015). Indeed, the extent of neurotoxicity associated with OPC extends beyond AChE inhibition. They can provoke inflammatory and immunological responses that contribute to both acute and chronic neurological impairments. The current study uncovered that malathion could disrupt the delicate balance between pro-inflammatory and anti-inflammatory immune responses by affecting the key transcription factors RORγt and FOXP3. This imbalance may contribute to immune dysregulation and increased susceptibility to inflammatory and immunological reactions.

In the present study, the gene expressions of RORγt and STAT3 were elevated in the malathion-intoxicated group, along with marked elevation in the pro-inflammatory cytokines IL-17 and IL-22. RORγt stimulated the expression of Th17-associated cytokines, such as IL-17 and IL-22, thus fostering the pro-inflammatory reactions. Furthermore, STAT3, which is responsible for the differentiation of Th17 cells, where STAT3 incorporation with RORγt regulated the expression of IL-17 and IL-22, resulting in inflammatory reactions (Kumar et al. 2021; Wu et al. 2022; Chang et al. 2019). Previous studies revealed that RORγt aggravates neuroinflammation associated with colitis, thus contributing to neurobehavioral disorders. Zhang et al. declared that counteracting the elevated RORγt levels improved the learning and memory ability in Alzheimer’s rats (Zhang et al. 2015). Oliva et al. reported that astrocytes respond to trauma by stimulating inflammatory signaling mediated by STAT3 (Oliva et al. 2012). Chen and his coworkers demonstrated that the suppression of STAT3 during brain inflammation would inhibit astrogliogenesis and promote neurogenesis (Chen et al. 2013). These findings pinpoint that the RORγt/STAT3 axis, which triggers the pro-inflammatory cytokines IL-17/IL-22, could be implicated in promoting neuroinflammation induced by OPC. These elucidations are supported by literature that revealed that OPC triggers the immunological disturbance (Lou, et al. 2020).

In parallel, the current study revealed that the anti-inflammatory axis FOXP3/STAT5/IL-10 was markedly inactivated in the malathion-intoxicated group, where the gene expressions of FOXP3 and STAT5 were suppressed along with a reduced level of IL-10. FOXP3 is a transcription factor that is essential for the differentiation of Tregs, which governs the expression of genes involved in the production of anti-inflammatory cytokines like IL-10. Besides, STAT5 translocates to the nucleus and promotes the transcription of FOXP3, thereby facilitating Treg development and maintenance (Passerini et al. 2008; Wang et al. 2008; Tsuji-Takayama et al. 2008). Dansokho et al. showed that transient depletion of Treg cells accelerated the onset of cognitive deficits and was associated with modulation of the microglial response to deposits of Aβ peptide. Modulating the function of Tregs has been proposed as a potential therapeutic option to mediate neuroprotection in neuroinflammation-mediated disorders, including Parkinson’s disease and amyotrophic lateral sclerosis (Reynolds et al. 2007; Beers et al. 2011). Furigo et al. indicated that the brain STAT5 signaling is required to attain normal learning and memory (Furigo et al. 2018). These findings highlight the ability of malathion to impair STAT5 signaling and reduce FOXP3 anti-inflammatory potential as well as suppress the levels of IL-10, thus shifting the balance towards pro-inflammatory responses in the CNS, as demonstrated in the current study. Worthy noted that IL-10 is an essential cytokine in the brain in order to ensure nervous tissue survival and mitigate inflammatory responses, thus triggering several signaling pleiotropic pathways (Strle et al. 2002).

Altogether, these findings disclosed that malathion exerts its deleterious effects by disturbing the balance between pro- and anti-inflammatory mechanisms, favoring chronic inflammation. This was more evidenced by the low ratio between FOXP3/RORγt, along with elevated ratios of STAT3/STAT5 and IL-17/IL-10, compared with the control group. The immune system maintains a dynamic equilibrium between pro-inflammatory and anti-inflammatory mediators. A shift towards inflammation can lead to tissue damage, oxidative stress, and neuroinflammation. A balanced response is crucial for recovery, repair, and avoidance of chronic inflammation or immune suppression. A reciprocal relationship has existed between Th17 and Treg cell types. FOXP3, the master regulator of Treg development, and ROR-γt, the key regulator of Th17 differentiation, have been shown to antagonize each other. Several factors are interplayed to determine whether the immune response is skewed towards Th17 cells or Treg cells (Koenen et al. 2008; Zhou et al. 2008). Thus, malathion, through its toxic effects, could disrupt this immune homeostasis as prevailed in the current study.

Over and beyond, IL-1β was highly expressed in the malathion-intoxicated group, reflecting the exacerbation of inflammation, which could lead to increased blood-brain barrier permeability and amplify the release of other cytokines (Versele et al. 2022). Ample evidence revealed the link between IL-1β and FOXP3/RORγt, where IL-1β suppresses Treg development by downregulating FOXP3 expression, promoting an environment unfavorable for Treg stability (Mailer et al. 2015; Deknuydt et al. 2009). On the contrary, IL-1β, especially in combination with other cytokines, drives naive CD4+ T cells to differentiate into Th17 cells via the upregulation of RORγt (Cella et al. 2010).

In addition to the aforementioned findings, the malathion-intoxicated group exhibited activated apoptosis mechanisms, which were indicated by the elevation of the apoptotic marker BAX and the reduction of the antiapoptotic marker BCL2. This apoptotic cascade could be triggered by the previously mentioned immunological and inflammatory disturbances (Bamberger and Landreth 2002). FOXP3 and RORγt regulate more than just immune cells’ fate, but they also influence cell survival, where FOXP3 protects against apoptosis by reducing inflammation, while RORγt promotes apoptosis indirectly through inflammatory cytokines. Their balance is a checkpoint between immune activation and tissue damage. This is supported by Chu et al., who revealed that the inhibition of FOXP3 promotes apoptosis (Chu et al. 2015). Indeed, apoptotic cell death plays an important role in the inflammatory processes and in the resolution of inflammatory reactions (Griffiths et al. 2009).

The BCG vaccine has attracted a lot of interest as a promising immunological regulator with potential therapeutic applications beyond infectious diseases. Its potential in reshaping the immune landscape has gained interest in the field of neuroinflammatory issues (Bamberger and Landreth 2002; Jurczak and Druszczynska 2025). BCG promoted the regulatory immune responses, via enhancing FOXP3 and its associated anti-inflammatory cytokines such as IL-10, while suppressing pro-inflammatory effects associated with RORγt and IL-17, hence restored the immunological homeostasis and could oppose the ongoing inflammation accompanied with neurological impairments (Yedke and Kumar 2024). Moreover, BCG exhibited neuroprotective effects in preclinical models of multiple sclerosis and other CNS diseases, via regulating the microglial and astrocyte activation. Owing to its safety margin and immunomodulatory properties, BCG could be a promising therapeutic agent for conditions characterized by immune dysregulation and neuroinflammation as in malathion exposure (Cossu et al. 2022).

Intriguingly, the present study showed that the administration of BCG was able to markedly ameliorate the stimulation of the proinflammatory RORγt/STAT3/IL-17/IL-22 axis, where the gene expression of RORγt and STAT3 were markedly lower, along with markedly lower levels of IL-17 and IL-22 in the BCG-treated group compared with the non-treated one. These findings are in alignment with Khader et al., who found that the low-frequency Th17 cells were important for immune protection from *Mycobacterium tuberculosis* (Khader et al. 2007), as well as Jaron et al., who revealed the reduced capacity of Th17 cells to produce IL-17 following immunization (Jaron et al. 2008). Further, IL-17 can promote tissue damage in the context of other infectious and autoimmune diseases (Khader et al. 2007; Miossec and Kolls 2012; Korn et al. 2009; Diveu et al. 2008; Kozakiewicz et al. 2013). On the contrary, the present study showed that BCG administration markedly augmented the anti-inflammatory FOXP3/STAT5/IL-10 axis, where the gene expression of FOXP3 and STAT5 were elevated along with increased content of IL-10 in the BCG-treated group compared with the non-treated one. This comes in agreement with Keefe et al. who stated that BCG restores immune balance through Treg induction, which is indicated by elevated FOXP3 (Hori et al. 2003; Keefe et al. 2021). Villaseñor et al. concluded that BCG promotes IL-10 expression (Villasenor et al. 2019). The present study documented that BCG possessed immunomodulation and anti-inflammatory activities, which could aid in mitigating the deleterious effects associated with malathion exposure.

Preceding studies highlighted the multifaceted functions of BCG for managing diverse diseases. Zuo et al. revealed that BCG treatment improved cognitive function and alleviated neuroinflammation via reducing pro-inflammatory cytokines, and increased anti-inflammatory cytokines in the Alzheimer’s model (Zuo et al. 2017). Another study showed that neonatal BCG vaccination alleviated sickness, anxiety, and depression-like behavior and reduced hippocampal cell proliferation impairments and pro-inflammatory responses triggered by lipopolysaccharide exposure in adulthood (Yang et al. 2016). Similarly, the current study revealed that BCG mitigates the neurobehavioral deficits induced by malathion. In addition, the BCG-treated group showed a better performance in anxiety, depression, cognition, and locomotor behavioral tests, reflecting that BCG offers neuroprotective benefits by mitigating neuroinflammation and behavior impairments. Over and above, the histopathological examinations complemented these findings, where the cerebral cortex of the BCG-treated rats showed a significant tissue improvement. Most neurons were normal with clear vesicular nuclei, while a few were apoptotic with shrunken, hyperchromatic nuclei. All of these findings reflect BCG’s capacity as a prophylactic agent in diseases driven by immune and inflammatory dysregulations. Besides, the anti-inflammatory effects of BCG were extended to IL-1β and GFAP, where their expressions were lower in the BCG group.

In the same paradigm, the apoptotic marker BAX was decreased together with a high level of the anti-apoptotic marker BCL2 among BCG-treated rats. In neuroinflammation, BCG could regulate apoptosis by modulating the immune responses to hinder excessive neuronal cell death. Yedke et al. showed that BCG treatment reversed motor abnormalities, reduced oxidative stress and neuroinflammatory markers, apoptotic mediators, and striatal lesions in quinolinic acid-induced neurotoxicity (Yedke et al. 2023). The anti-apoptotic neuronal effects revealed in the current study could be attributed to BCG’s capability to attenuate excessive inflammation, thereby reducing apoptosis of neurons and glial cells. This effect is likely linked to the previously mentioned increase in anti-inflammatory cytokines like IL-10 and FOXP3 that counterbalance the pro-inflammatory signal of RORγt and its associated inflammatory cytokines. Contrarily, previous studies about cancer revealed that BCG could exert an apoptotic effect on cancer cells (Schwarzer et al. 2010). These swings in BCG’s activity could be ascribed to cell type**,** disease context**,** and immune status of the host.

Additionally, the current study compared the efficiency of BCG against scopolamine, a well-characterized central muscarinic antagonist used here as a reference anticholinergic agent. Both BCG and scopolamine showed similar behavioral effects, whereas BCG showed superior effects in improving histological alterations compared with scopolamine. Regarding the molecular and biochemical investigations, the repercussions of scopolamine on attenuating the pro-inflammatory arm RORγt/STAT3/IL-17/IL-22 were equivalent to BCG efficiency. However, with regard to the anti-inflammatory arm FOXP3/STAT5/IL-10, BCG showed a higher increase of FOXP3 expression than scopolamine. Regarding the apoptotic markers, both BCG and scopolamine showed comparable effects. However, scopolamine mainly functions as an antagonist of muscarinic receptors, which modulates neuroinflammation by acting on the cholinergic system. It has no direct immunomodulatory effects, although it might have some downstream effects on immunological responses via the cholinergic anti-inflammatory system. Another difference between them is that while scopolamine provides temporary symptom alleviation, BCG has a longer-lasting impact.

## Conclusions

The current study emphasized the deleterious effects of OPC exposure on the perturbations of the neuroimmune homeostasis. Malathion triggered neuroinflammation via cholinergic stimulation as well as distorted the equilibrium between the proinflammatory (RORγt/STAT3/IL-17/IL-22) and the anti-inflammatory (FOXP3/STAT5/IL-10) signaling. This imbalance contributes to fostering cytokine release, neuronal apoptosis, and neurodegeneration. Importantly, BCG showed marked effects in mitigating these neurotoxic effects via sustaining the immune equilibrium, decrement pro-inflammatory mediators, and promoting anti-inflammatory and neuroprotective effects. These findings indicated a promising immunotherapeutic role for of BCG in the counteraction of OPC-induced neurotoxicity. Future studies is warranted to confirm the mechanistic link by employing selective STAT3/STAT5 inhibitors or agonists as well as to translate these findings to the clinical settings. Both ACh and AChE assessments to more definitively characterize cholinergic disruption induced by malathion.

## Data Availability

The datasets used and/or analyzed during the current study are available from the corresponding author on reasonable request.
